# Reducing Systematic Centroid Errors Induced by Fiber Optic Faceplates in Intensified High-Accuracy Star Trackers

**DOI:** 10.3390/s150612389

**Published:** 2015-05-26

**Authors:** Kun Xiong, Jie Jiang

**Affiliations:** Key Laboratory of Precision Opto-Mechatronics Technology, Ministry of Education, School of Instrumentation Science and Opto-Electronics Engineering, Beijing University of Aeronautics and Astronautics (BUAA), Beijing 100191, China; E-Mail: xiongkun8748@163.com

**Keywords:** intensifier, fiber optic faceplate, intensified star tracker, systematic centroid error

## Abstract

Compared with traditional star trackers, intensified high-accuracy star trackers equipped with an image intensifier exhibit overwhelmingly superior dynamic performance. However, the multiple-fiber-optic faceplate structure in the image intensifier complicates the optoelectronic detecting system of star trackers and may cause considerable systematic centroid errors and poor attitude accuracy. All the sources of systematic centroid errors related to fiber optic faceplates (FOFPs) throughout the detection process of the optoelectronic system were analyzed. Based on the general expression of the systematic centroid error deduced in the frequency domain and the FOFP modulation transfer function, an accurate expression that described the systematic centroid error of FOFPs was obtained. Furthermore, reduction of the systematic error between the optical lens and the input FOFP of the intensifier, the one among multiple FOFPs and the one between the output FOFP of the intensifier and the imaging chip of the detecting system were discussed. Two important parametric constraints were acquired from the analysis. The correctness of the analysis on the optoelectronic detecting system was demonstrated through simulation and experiment.

## 1. Introduction

Star trackers are widely used in the attitude measuring systems of stationary satellites because of their advantages, which include high accuracy, non-drifting feature and automation [1,2,3]. Besides stationary applications, strategic missiles, high-maneuver satellites, *etc.* also employ star trackers [4,5] to provide high accuracy attitude information for gaining control. However, the star spots in an image will be dragged into the strips during maneuvering, or in worse cases, the strips will be so weak that they will be submerged in background noise.

Consequently, numerous discussions and studies [6,7] have been conducted on parameter optimization in optoelectronic detection systems to enhance their dynamic performance. Some researchers have claimed that the impact of maneuvering can be compensated by setting a relevant short period of exposure time followed by processing [8], while others employed optical lenses with large field of view (FOV) to observe more stars. Either short exposure or large FOV can improve the dynamic performance in some degree by means of tradeoffs. However, both tactics would cause decreasing sensitivity of optoelectronic systems as side effects. As a result, stars may not be observed by the star trackers employing these tactics when the maneuvering angular speed is high (5° per second or more). Revolutionarily, low light-level detectors with astounding increases in sensitivity were introduced as new-generation imaging devices for star trackers. Among such detectors, the intensified complementary metal oxide semiconductor (ICMOS) and the intensified charge coupled device (ICCD) exhibit the advantages of high sensitivity, small volume and low power consumption. In addition, these detectors have no requirement for extra cooling. Such advantages make ICMOS and ICCD ideal as imaging devices for the optoelectronic systems of star trackers. The intensifier consists of multiple FOFPs, hence, the optoelectronic detecting system is complicated and may cause accuracy loss in attitude measurement.

To date, a considerable number of analyses [9,10,11,12,13] have been performed on the influence of the intensifier on imaging. Given the traditional applications of the intensifier in night vision, the modulation transfer function (MTF), which describes the capability of a device to transfer different spatial frequency signals, is regarded as the key characteristic of optoelectronic systems. However, the performance of high-accuracy star trackers relies mainly on the accurate observation of starlight vectors derived from the centroid positions of star spots [14]. Compensation of systematic centroid errors in normal star trackers carried out in the frequency domain achieves sound results [15], while few studies on systematic centroid errors in the optoelectronic detecting system of intensified star trackers have been conducted. In Section 2, the imaging principle of the optoelectronic detecting system is described, and error sources related to FOFP are analyzed. In Section 3, a general expression of the systematic centroid error from an arbitrary optoelectronic component is obtained through centroid localization analysis in the frequency domain. Given that FOFP functions as the basic component of the intensifier, its image transmission model is presented in Section 4. Based on the analysis of FOFP, three FOFP-induced systematic error sources in the optoelectronic system for intensified star trackers are discussed in Section 5 to minimize possible errors that they could cause. Two parametric constraints are deduced from the discussion. The simulations and experiments presented in Section 6 validate the analysis made in this study.

## 2. Optoelectronic Detecting System of Intensified High-Accuracy Star Trackers

The imaging principle of the optoelectronic system is described in this section, particularly the low light-level enhancement mechanism in the intensifier. Different error sources related to FOFPs are analyzed, and FOFP-induced systematic centroid error is classified into three types.

### 2.1. Imaging Principle

The optoelectronic system of intensified high-accuracy star trackers works as follows: approximately infinitely far starlight enters the optical lens as parallel light. Passing through the lenses, the starlight converges into a Gaussian spot. Weak star spots enter the intensifier, and the intensity of the spots is enhanced. Complementary metal oxide semiconductor (CMOS) or charge coupled device (CCD) imaging chips coupled at the rear of the intensifier convert analog image signals into discrete digital image signals, which are transferred into the information processing system of the star tracker.

**Figure 1:**
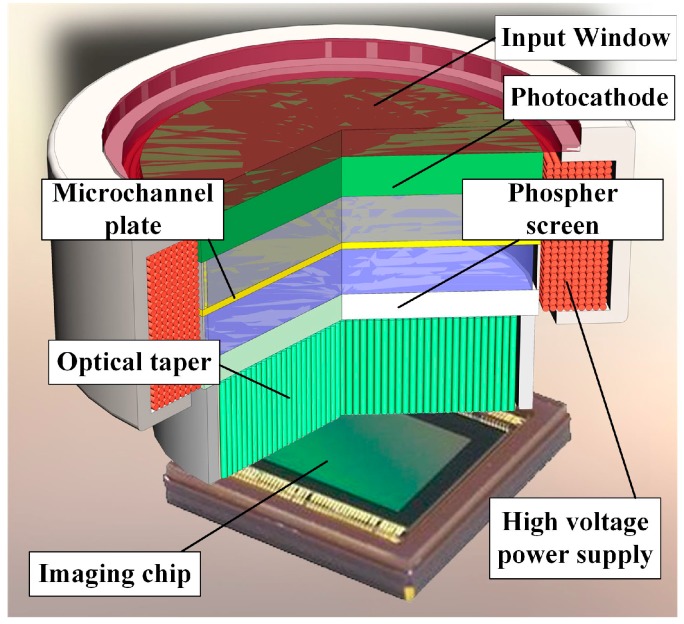
Internal structure of the intensifier [16].

The core component of the detecting system is the intensifier. The intensified star tracker employed in this study uses the Generation II+ double-proximity focusing image intensifier, and its internal structure is shown in Figure 1. The photocathode supplies primary photoelectrons to the input face of the micro-channel plate (MCP) through its chemical coating, which transforms optical signals into electric signals. A few photoelectrons enter the MCP and are multiplied under the effect of the high-potential electric field. The secondary electrons from the MCP strike a phosphor screen that emits photons.

### 2.2. FOFP-Induced Centroid Error

Both the photocathode and the anode of the intensifier normally bond to an FOFP. Similar to an FOFP, the MCP also arranges its micro-channels hexagonally, and thus, it is regarded as the equivalent of FOFP for electrons. In addition, the photons emitting from the screen are optically coupled to the imaging chips by an FOFP, which is called an optical taper. That is, it is the intensifier that is a compound with multiple FOFPs. For intensified star trackers, addition of an image intensifier will cause complex centroid errors. Firstly, the gain variation attributed to the quantum effect in the electron multiplying process of MCP causes centroid errors that change over time. Secondly, the defection of fiber arrangement in FOFP causes centroid errors that change with position. These two error sources cannot be described by specific expressions, and thus, they are difficult to correct or reduce. Thirdly, given that FOFP is actually a discrete optical component that is neither isoplanatic nor shift invariant, it can cause systematic centroid errors that are distinct at different places.

**Figure 2:**
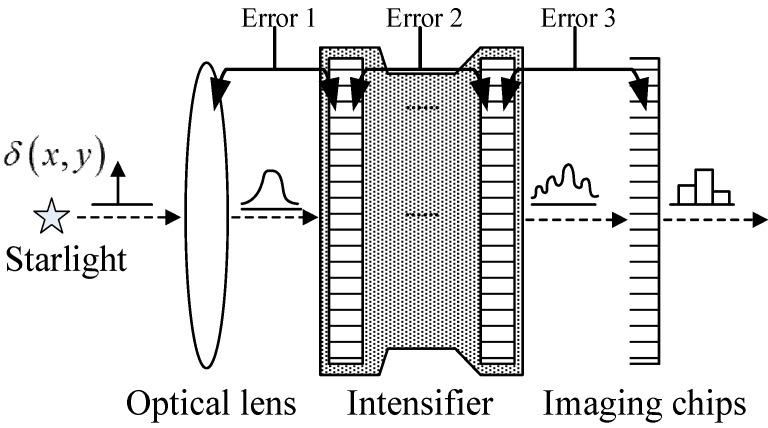
Diagram of the FOFP-induced systematic centroid errors.

In an optoelectronic detecting system, the intensifier is located between the optical lens and the imaging chip, as shown in Figure 2. Three error sources are found along the entire optical path when the systematic centroid errors of an FOFP are involved: Error 1: Systematic errors occur when the star spot from the optical lens enters the input FOFP of the intensifier. Error 2: The multiple FOFPs of the intensifier will lead to even more complex systematic errors. Error 3: The star spot that is emitting from the output FOFP of the intensifier enters imaging chip coupling from behind and generates systematic errors because of the sampling of the imaging chip [17]. The errors caused by the aforementioned sources are collectively referred to as FOFP-induced systematic centroid errors in this study. All these errors are covered by the following investigation.

## 3. Frequency Domain Analysis of Centroid Localization

The direct error analysis of a sophisticated optoelectronic system can be complicated, and thus, frequency domain analysis is a better alternative. The expressions of centroid localization and the centroid error of an arbitrary optical component are presented in this section and function as the basis for the following discussion on FOFP-induced systematic centroid error.

### 3.1. Centroid Localization Expression in the Frequency Domain

The subdivided centroid localization methods for star trackers can be generally divided into the mass center method and the surface fitting method. To improve star spot position estimation, several new methods consider the weight functions or change the order of the moment in the mass center method, whereas others change the surface expression in the surface fitting method. When real time is considered, the mass center method has been widely used because of its advantages of small computational cost and easily realized algorithm structure. The discussion on centroid error in this paper is specific to the mass center method.

The mass center method in centroid localization assumes that the exact location of a star spot coincides with its mass center of luminance. Given that an image is discretized into pixels by the imaging sensor, the mass center method is always expressed in summation form. More generally in the continuous spatial domain, by denoting the intensity distribution of a star spot as *f*(*x*,*y*), the centroid location of the spot is given as follows:

(1) $$\bar{x}=\frac{\iint xf(x,y)\mathrm{dxdy}}{\iint f(x,y)\mathrm{dxdy}},\bar{y}=\frac{\iint yf(x,y)\mathrm{dxdy}}{\iint f(x,y)\mathrm{dxdy}}$$

where *x* and *y* are the metrics of two orthogonal spatial axes. By applying Fourier transform to the distribution function *f*(*x*,*y*), its frequency domain expression is given as follows:

(2) $$F\left(u,v\right)=\int_{-\infty }^{+\infty }\int_{-\infty }^{+\infty }f\left(x,y\right)\exp\left[-j2\pi \left(ux+vy\right)\right]\text{dxdy}$$

where *u* and *v* are the frequency metrics that correspond to two orthogonal spatial axes. Deducing that the partial derivative of *F*(*u*,*v*) with respect to *u* is expressed as follows:

(3) $$F_{u}\left(u,v\right)=\frac{\partial F}{\partial u}=-j2\pi \cdot \int_{-\infty }^{+\infty }\int_{-\infty }^{+\infty }xf\left(x,y\right)\exp\left[-j2\pi \left(ux+vy\right)\right]\text{dxdy}$$

In the origin of the frequency plane, *F*(*u*,*v*) and its partial derivative *F_u_*(*u*,*v*) can be simplified as follows:

(4) $$F\left(0,0\right)=\int_{-\infty }^{+\infty }\int_{-\infty }^{+\infty }f\left(x,y\right)\exp\left[-j2\pi \left(0x+0y\right)\right]\text{dxdy=}\int_{-\infty }^{+\infty }\int_{-\infty }^{+\infty }f\left(x,y\right)\text{dxdy}$$

(5) $$F_{u}\left(0,0\right)\text{=}-j2\pi \cdot \int_{-\infty }^{+\infty }\int_{-\infty }^{+\infty }xf\left(x,y\right)\text{dxdy}$$

By comparing the two preceding expressions with the expression of the mass center method in the spatial domain (shown in Equation (1)), we obtain:

(6) $$\frac{F_{u}\left(0,0\right)}{F\left(0,0\right)}=\frac{-j2\pi \cdot \int_{-\infty }^{+\infty }\int_{-\infty }^{+\infty }xf\left(x,y\right)\text{dxdy}}{\int_{-\infty }^{+\infty }\int_{-\infty }^{+\infty }f\left(x,y\right)\text{dxdy}}=-j2\pi \bar{x}$$

The expression is simplified as follows:

(7) $$\bar{x}=\frac{F_{u}\left(0,0\right)}{-j2\pi F\left(0,0\right)}$$

Equation (7) associates the centroid location with the spectrum expression of the spot in the frequency domain. According to the equation, the centroid location is only related to the origin point values of the star spot spectrum in the frequency domain and its partial derivative.

### 3.2. Systematic Centroid Error Expression

Assuming that the spot pattern goes through a shift-invariant optoelectronic component, the Point Scattering Function (PSF) of the part is denoted as *h*(*x*,*y*), and the distribution of the output image *g*(*x*,*y*) is given as follows:

(8) g(x,y)=f(x,y)∗h(x,y)

In the frequency domain, the course can be expressed as a direct multiplication instead of a convolution as follows:

(9) G(u,v)=F(u,v)⋅H(u,v)

From Equation (7), the centroid location that is going through the preceding system will change as follows:

(10) $$\tilde{x}=\frac{G_{u}\left(0,0\right)}{-j2\pi G\left(0,0\right)}=\frac{F_{u}\left(0,0\right)H\left(0,0\right)+F\left(0,0\right)H_{u}\left(0,0\right)}{-j2\pi F\left(0,0\right)H\left(0,0\right)}=\bar{x}+\frac{H_{u}\left(0,0\right)}{-j2\pi H\left(0,0\right)}$$

Thus, the systematic centroid of the component is as follows:

(11) $$\text{Δ}x=\tilde{x}-\bar{x}=\frac{H_{u}\left(0,0\right)}{-j2\pi H\left(0,0\right)}$$

According to Equation (11), when starlight comes through an optoelectronic component, the involved centroid error only depends on the system itself. Furthermore, for an isoplanatic component (which is also translation invariant), it is easy to infer that the systematic error it brings about is constant in the image plane.

However, in a shift-variant system, PSF varies from place to place throughout the image plane. PSF is denoted as $h{|}_{\left(\bar{x},\bar{y}\right)}\left(x,y\right)$, where $\left(\bar{x},\bar{y}\right)$ represents the location of the input spot *f*(*x*,*y*). Meanwhile, its expression in the frequency domain is denoted as $H{|}_{\left(\bar{x},\bar{y}\right)}\left(u,v\right)$. Following Equation (11), the systematic error of the shift-variant system is given as follows:

(12) $$\text{Δ}x_{\left(x_{c},y_{c}\right)}=\frac{H_{u}{|}_{\left(x_{c},y_{c}\right)}\left(0,0\right)}{-j2\pi H{|}_{\left(x_{c},y_{c}\right)}\left(0,0\right)}$$

According to the preceding equation, the systematic centroid error in the shift-variant system varies with the position of the input object. In particular, the discrete optic component has microcells that repeat periodically in certain spatial structures, and thus, the systematic error that it causes is also periodic.

## 4. Image Transmission Model of FOFP

**Figure 3:**
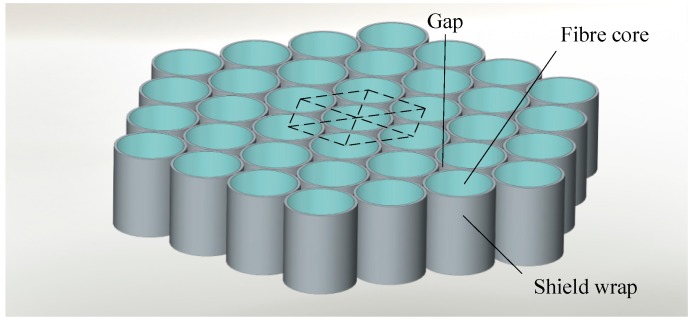
Diagram of FOFP structure.

FOFP is a common image-carrying component that consists of a huge amount of fibers arranged in a hexagonal grid structure similar to a honeycomb. It is optically equivalent to a zero-thickness window. As mentioned in Section 2, an intensifier mainly consists of multiple FOFPs; hence, analyzing FOFP is highly important. A diagram that depicts FOFP structure is shown in Figure 3.

**Figure 4:**
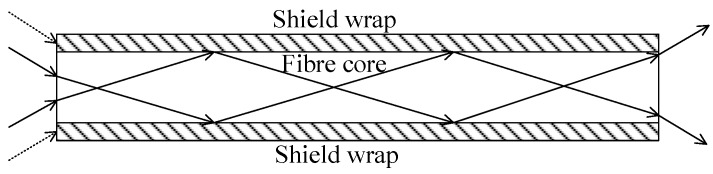
Cutaway diagram of a single fiber.

A cutaway diagram of a single fiber is shown in Figure 4. The incident photons that are hitting the end face of the shield wrap will not get through (shown in dotted arrows), whereas those entering the fiber core will pass through via total reflection (shown in solid arrows). The fibers are carefully arranged to ensure that image transmission will not have any distortion. A lightproof shield wrap that surrounds the fiber core is used to block cross disturbance among fibers. In addition, fibers are typically cylindrical; therefore, small gaps exist between neighboring fibers because of the hexagonal arrangement of the structure. Neither the shield wrap nor the small gaps can transmit light. Consequently, these dead zones in the optic fiber plate will affect both the imaging and the centroid localization of high-accuracy star trackers.

Disregarding its thickness, absorption loss, transmission spectrum *etc.*, an FOFP can be regarded as a mask of round holes with a particular arrangement. When an image is passing through an FOFP, it follows the processes of integration, sampling and reimaging in order. In the first two steps, the light entering an FOFP converges its intensity in the fiber cores and forms energetic impulses. In reimaging, the energy pulse emerges from the fiber core in a certain pattern. The PSF of FOFP *h*(*x*,*y*) can be expressed as follows:

(13) $$h\left(x,y\right)=h_{samp}\left(x,y\right)*h_{re}\left(x,y\right)$$

The input end face of a single fiber is a circle, and thus, the integration process can be described by cylinder function circ(*r*) as follows:

(14) $$\text{circ}(r)=\text{circ}(\sqrt{x^{2}+y^{2}})=\{\begin{array}{cc} 1 & r\leq 1\\ 0 & r>1 \end{array}$$

**Figure 5:**
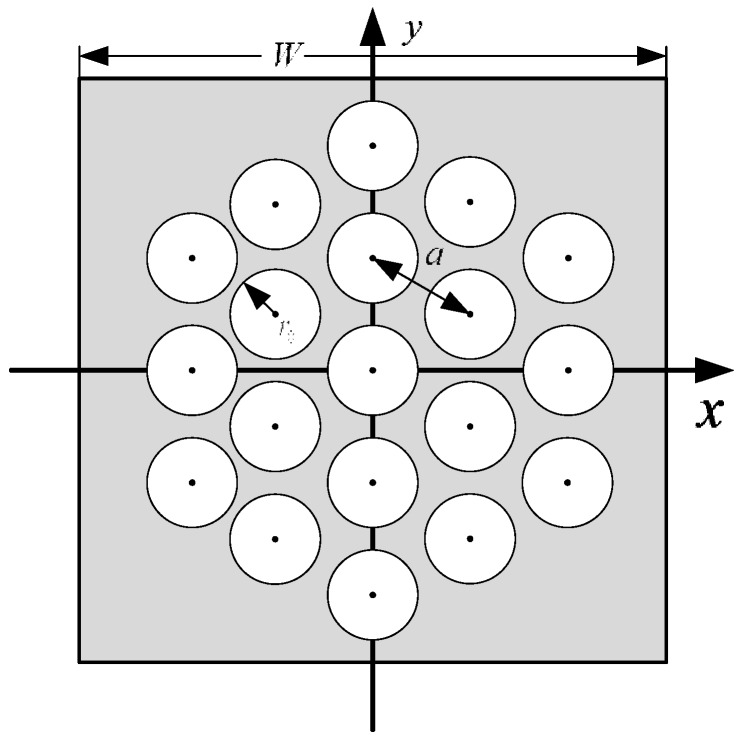
FOFP model.

A diagram of the ‘mask plate’ that represents the FOFP mechanism is provided in Figure 5, where the radius of the fiber core is denoted as *r*_0_ and the fiber interval is *a*. The arrangement of the fibers is shown in the figure, and the size of the FOFP is a square of *W* × *W*. Then, the integration–sampling function of the FOFP can be expressed as follows:

(15) $$h_{samp}\text{=}\left[\text{circ}\left(\frac{r}{r_{0}}\right)\cdot \text{comb}\left(\frac{x}{\sqrt{3}a}+\frac{y}{a},\frac{x}{\sqrt{3}a}-\frac{y}{a}\right)\right]\cdot \text{rect}\left(\frac{x}{W},\frac{y}{W}\right)$$

The reimaging process is complicated. Reimaging varies with different optical paths inside the fiber caused by various incident angles and incident positions of the incident light. Under ideal circumstances, the energy is believed to output fibers uniformly. Hence, the reimaging function can be written as follows:

(16) $$h_{re}=\text{circ}\left(\frac{r}{r_{0}}\right)$$

According to Equations (13)–(16), the PSF of an FOFP can be expressed as follows:

(17) $$h\left(x,y\right)=\left[\text{circ}\left(\frac{r}{r_{0}}\right)\cdot \text{comb}\left(\frac{x}{\sqrt{3}a}+\frac{y}{a},\frac{x}{\sqrt{3}a}-\frac{y}{a}\right)\cdot \text{rect}\left(\frac{x}{W},\frac{y}{W}\right)\right]*\text{circ}\left(\frac{r}{r_{0}}\right)$$

where $r=\sqrt{x^{2}+y^{2}}$.

The Fourier transform of the PSF is the modulation transfer function (MTF), *i.e.*,:

(18) $$H\left(u,v\right)=\left[\frac{J_{1}\left(2\pi r_{0}f\right)}{\pi r_{0}f}*\text{comb}\left(\frac{\sqrt{3}a}{2}u+\frac{a}{2}v,\frac{\sqrt{3}a}{2}u-\frac{a}{2}v\right)\text{*sinc}\left(Wu,Wv\right)\right]\cdot \frac{J_{1}\left(2\pi r_{0}f\right)}{\pi r_{0}f}$$

where $f=\sqrt{u^{2}+v^{2}}$. Given that the size of an FOFP is significantly greater than the scale of a fiber, the frequency transform of the rect function, *i.e.*, sinc(*Wu*,*Wv*), can be considered as a unit of impulse function, as follows:

(19) $$\underset{W\to \infty }{\lim}\text{sinc}\left(Wu,Wv\right)=\delta \left(u,v\right)$$

Then, the MTF of an FOFP becomes:

(20) $$H\left(u,v\right)=\left[\frac{J_{1}\left(2\pi r_{0}f\right)}{\pi r_{0}f}*\text{comb}\left(\frac{\sqrt{3}a}{2}u+\frac{a}{2}v,\frac{\sqrt{3}a}{2}u-\frac{a}{2}v\right)\right]\cdot \frac{J_{1}\left(2\pi r_{0}f\right)}{\pi r_{0}f}.$$

## 5. Reducing FOFP-Induced Systematic Centroid Errors

As discussed in Section 2.2, three types of FOFP-induced systematic centroid errors occur in the optoelectronic detecting system of intensified star trackers. Basically, each error is determined by the match between a component and the succeeding one. The matches are discussed by means of a frequency spectrum. The suppression of all the aforementioned error sources is analyzed chronologically in this section.

### 5.1. Systematic Error Reduction between the Optical Lens and the Input FOFP of an Intensifier

#### 5.1.1. Optical Lens Analysis

Theoretically, starlight distribution before the optical lens can be regarded as an energetic impulse function *Eδ*(*x*−*x*_0_, *y*−*y*_0_). When the lenses are modulated, the impulse point blurs into a spot. Assuming that the lenses are equivalent to an ideal convex lens, and the PSF of the lens *h*_0_(*x*, *y*) will be the same Gaussian function everywhere, as follows:

(21) $$h_{0}\left(x,y\right)=\frac{1}{2\pi \sigma^{2}}e^{\frac{\text{-}\left(x^{2}+y^{2}\right)}{2\sigma^{2}}}$$

where 3σ is the radius of the spot. The MTF of the lenses is as follows:

(22) $$H_{0}\left(u,v\right)=e^{-2\pi^{2}\sigma^{2}\left(u^{2}+v^{2}\right)}$$

Then, the star spot behind the lenses can be written as follows:

(23) $$f\left(x,y\right)=\left[E\delta \left(x-x_{0},y-y_{0}\right)\right]*h_{0}\left(x,y\right)=\frac{E}{2\pi \sigma^{2}}e^{-\frac{{\left(x-x_{0}\right)}^{2}+{\left(y-y_{0}\right)}^{2}}{2\sigma^{2}}}$$

The ideal optical lens is translation invariant; hence, it has no effect on centroid localization according to Equation (10), as follows:

(24) $$\text{Δ}x=\frac{F_{u}\left(0,0\right)}{-j2\pi F\left(0,0\right)}=0,\text{Δ}y=\frac{F_{v}\left(0,0\right)}{-j2\pi F\left(0,0\right)}=0$$

That is, centroid localization will remain error-free after passing through the lenses.

#### 5.1.2. Systematic Centroid Error of an FOFP

According to the previous analysis, the effect of an FOFP on the input image can be divided into three separate processes: integration, sampling and reimaging. The reimaging process is a translation-invariant cylinder function (as shown in Equation (16)). According to the analysis in Section 3.2, no systematic error will be evolved by the reimaging process. That is, the systematic centroid error caused by an FOFP is formed during integration and sampling.

**Figure 6:**
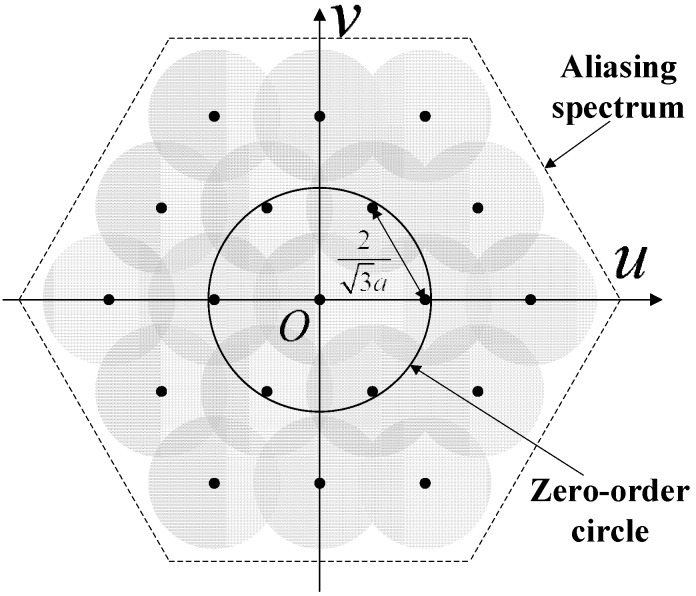
Overlapping frequency points in the frequency domain.

In the integration and sampling processes, the input image initially convolves with the cylinder function circ(*r*), and then, the result is sampled by a hexagonal comb function. The transform of the spatial hexagonal comb function in the frequency domain is still a hexagonal comb function. Any image pattern that goes through the plate will have its frequency spectrum replicated and overlapped. The original frequency spectrum will be replicated to areas centered on each frequency point shown in the shaded area in Figure 6. These frequency points are assigned to a hexagonal grid structure that is perpendicular to one of the sample points in the spatial domain, with a distance of $2/\sqrt{3}a$ (approximately 1.15/*a*) between two neighboring points. These points are called overlapping frequency points.

The overlapping of the frequency spectrum can cause frequency aliasing. The effect of frequency aliasing on centroid localization is analyzed in this section. Assuming that starlight spot input in the FOFP obeys Gaussian distribution, the exact spot in the origin spot can be denoted as *f*_0_(*x*, *y*), and then, the distribution *f*(*x*, *y*) of a starlight spot in the arbitrary position $\left(\bar{x},\bar{y}\right)$ can be expressed as follows:

(25) $$f\left(x,y\right)=f_{0}\left(x-\bar{x},y-\bar{y}\right)$$

After integration, the PSF is convolved with the cylinder function as follows:

(26) $$g\left(x,y\right)=f\left(x,y\right)*\text{circ}\left(\frac{r}{r_{0}}\right)=f_{0}\left(x-\bar{x},y-\bar{y}\right)*\text{circ}\left(\frac{r}{r_{0}}\right)$$

After being transformed in the frequency domain, the Gaussian spectrum is multiplied by the first-order Bessel curved surface as follows:

(27) $$G\left(u,v\right)=F\left(u,v\right)\cdot \frac{J_{1}\left(2\pi r_{0}f\right)}{\pi r_{0}f}=e^{-j2\pi \left(u\bar{x}+v\bar{y}\right)}\cdot G_{0}\left(u,v\right)$$

where *G*_0_(*u*, *v*) represents the after-integration frequency spectrum of the star spot at the origin point. It would still be rotationally symmetric to the origin point in the frequency domain. After being transformed into frequency polar coordinates, spectrum *G*_0_(*u*, *v*) and its partial derivative can be expressed as follows:

(28) $$\{\begin{array}{c} \begin{array}{l} G_{0}\left(f,\theta \right)=G_{0}\left(f\right)\\ G_{0u}\left(f,\theta \right)=\cos\left(\theta \right)G_{0}^{\prime }\left(f\right) \end{array}\\ G_{0v}\left(f,\theta \right)=\sin\left(\theta \right)G_{0}^{\prime }\left(f\right) \end{array}$$

As shown in Figure 6, assuming that only the first-order overlapping spectra will interfere with the origin spectrum, then the aliasing spectrum $\tilde{G}\left(u,v\right)$ after the sampling process can be written as follows:

(29) $$\tilde{G}\left(u,v\right)=\sum_{n=-1}^{+1}G\left(u-nD,v\right)+\sum_{m=0}^{+1}\sum_{k=0}^{+1}G\left(u-mD-\frac{D}{2},v-\sqrt{3}kD-\frac{\sqrt{3}D}{2}\right)$$

where *D* represents the intervals between two overlapping frequency points (*i.e.*, $2/\sqrt{3}a$. According to Equations (28)–(29), we obtain

(30) $$\tilde{G}\left(0,0\right)=G_{0}\left(0\right)+2\left[\cos\left(2\pi D\bar{x}\right)+2\cos\left(\pi D\bar{x}\right)\cos\left(\sqrt{3}\pi D\bar{y}\right)\right]G_{0}\left(D\right)$$

(31) $${\tilde{G}}_{u}\left(0,0\right)=-j2\pi \bar{x}G\left(0,0\right)+2j{G_{0}}^{\prime }\left(D\right)\left[\sin\left(2\pi D\bar{x}\right)+\sin\left(\pi D\bar{x}\right)\cos\left(\sqrt{3}\pi D\bar{y}\right)\right]$$

(32) $${\tilde{G}}_{v}\left(0,0\right)=-j2\pi \bar{y}G\left(0,0\right)+2\sqrt{3}j{G_{0}}^{\prime }\left(D\right)\cos\left(\pi D\bar{x}\right)\sin\left(\sqrt{3}\pi D\bar{y}\right)$$

Compared with the first item in $\tilde{G}\left(0,0\right)$, the second one is significantly smaller. From Equation (12), the estimated centroid location under the aliasing influence of the first-order overlapping spectra can be approximately expressed as follows:

(33) $$\tilde{x}\approx \bar{x}-\frac{{G_{0}}^{\prime }\left(D\right)\left[\sin\left(2\pi D\bar{x}\right)+\sin\left(\pi D\bar{x}\right)\cos\left(\sqrt{3}\pi D\bar{y}\right)\right]}{\pi G_{0}\left(0\right)+2\pi \left[\cos\left(2\pi D\bar{x}\right)+2\cos\left(\pi D\bar{x}\right)\cos\left(\sqrt{3}\pi D\bar{y}\right)\right]G_{0}\left(D\right)}$$

(34) $$\tilde{y}\approx \bar{y}-\frac{\sqrt{3}{G_{0}}^{\prime }\left(D\right)\cos\left(\pi D\bar{x}\right)\sin\left(\sqrt{3}\pi D\bar{y}\right)}{\pi G_{0}\left(0\right)+2\pi \left[\cos\left(2\pi D\bar{x}\right)+2\cos\left(\pi D\bar{x}\right)\cos\left(\sqrt{3}\pi D\bar{y}\right)\right]G_{0}\left(D\right)}$$

The magnitude in frequency *D* is considerably less than that in the origin, and thus, the two preceding equations can be approximated as follows:

(35) $$\tilde{x}=\bar{x}-\frac{{G_{0}}^{\prime }\left(D\right)}{\pi G_{0}\left(0\right)}\left[\sin\left(2\pi D\bar{x}\right)+\sin\left(\pi D\bar{x}\right)\cos\left(\sqrt{3}\pi D\bar{y}\right)\right]$$

(36) $$\tilde{y}=\bar{y}-\frac{{G_{0}}^{\prime }\left(D\right)}{\pi G_{0}\left(0\right)}\sqrt{3}\cos\left(\pi D\bar{x}\right)\sin\left(\sqrt{3}\pi D\bar{y}\right)$$

The actual center of the input starlight spot is known as $\left(\bar{x},\bar{y}\right)$; hence, the systematic centroid error caused by the FOFP is as follows:

(37) $$\text{Δ}x\left(\bar{x},\bar{y}\right)=-\frac{{G_{0}}^{\prime }\left(D\right)}{\pi G_{0}\left(0\right)}\left[\sin\left(2\pi D\bar{x}\right)+\sin\left(\pi D\bar{x}\right)\cos\left(\sqrt{3}\pi D\bar{y}\right)\right]$$

(38) $$\text{Δ}y\left(\bar{x},\bar{y}\right)=-\frac{{G_{0}}^{\prime }\left(D\right)}{\pi G_{0}\left(0\right)}\sqrt{3}\cos\left(\pi D\bar{x}\right)\sin\left(\sqrt{3}\pi D\bar{y}\right)$$

**Figure 7:**
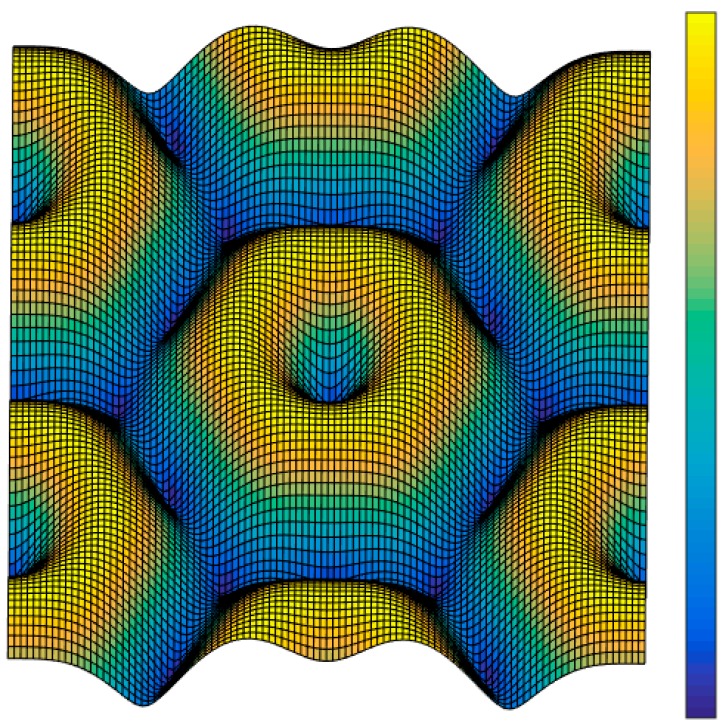
Magnitude of the systematic centroid error with respect to the entrance position of the starlight spot.

According to the result, the overall magnitude of the systematic error is determined by the ratio of the derivative value in the first-order overlapping points $G_{0}^{\prime }\left(D\right)$ to the spectrum value in the origin point *G*_0_(0). The repetition period of the systematic error is determined by the distance *D* between two neighboring overlapping frequency points. As shown in Figure 7, the systematic error magnitude distribution with starlight spots enters the FOFP from different places. If the spots are located at the center of a fiber or at the border of two neighboring fibers, then they would be affected by a minor systematic error. When the spots move from the border area and towards the fiber center, the error magnitude will initially increase and then decrease.

#### 5.1.3. Error Reduction

The integration process causes the Gaussian spot to become convoluted with the cylinder function. The Fourier transform of the cylinder function is the first-order Bessel function. Nearly all of the energy of this function is generally acknowledged to focus within the zero-order central circular area with a diameter of 1.22/*r*_0_. The sampling process will cause the original frequency spectrum to shift and overlap, with the interval of two neighboring overlapping frequency points being 1.15/*a*. Disregarding the thickness of the shield wrap (*r*_0_ = *a*), the diameter of the spectrum after integration becomes:

(39) $$1.22/r_{0}=1.22/a>1.15/a$$

If the thickness of the shield wrap is considered, then the spectrum diameter will be larger. In the analysis of the frequency domain, the accurate recovery of an image requires no aliasing in the spectrum after sampling. This condition is the essence of Shannon’s sampling theorem. However, the only requirement of a star tracker is that no systematic centroid error will result when an image passes through the component. According to the conclusion in Section 3.2, this condition indicates that the value at the origin point of the spectrum and its derivative value should not be affected by the component. As shown in Figure 6, the circle marks the minimum border of the zero-order central circle of the first-order Bessel function, within which lies six first-order overlapping shift points. Therefore, if no constraint is placed upon the star spot that is entering the input FOFP, then systematic error will be inevitable. The constraint to the spectrum of the entering image *F*(*u*,*v*) is as follows:

(40) $$F\left(u,v\right){|}_{f\geq 1.15/a}=0$$

The spectrum of the starlight spot from the lenses in the frequency domain still obeys Gaussian distribution. The star spot described by Equation (23) will have its frequency spectrum distribution within a circle that is 6*σ* in diameter. Thus, the upper frequency limit is as follows:

(41) $$f_{H}=3\cdot \frac{1}{2\pi \sigma }$$

By combining Equations (40) and (41), we obtain

(42) $$6\sigma \geq 6\cdot \frac{3}{2\pi }\cdot \frac{a}{1.15}\approx 2.49a$$

According to Equation (42), the circle of confusion (6*σ*) should not be less than 2.49 times that of the fiber interval to free the star tracker from systematic errors caused by the input FOFP.

### 5.2. Systematic Error Reduction among Multiple FOFPs

**Figure 8:**
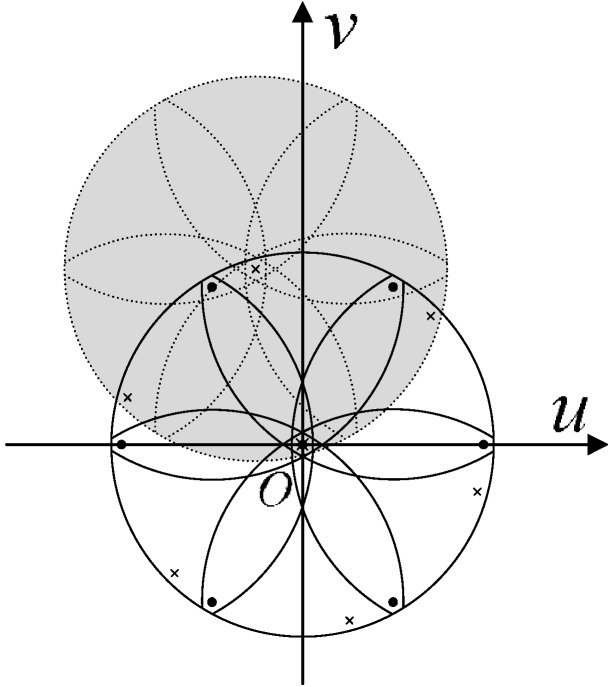
Frequency spectrum aliasing that causes centroid error.

In general, the intensifier contains more than one FOFP. This structure can cause more serious aliasing problems than that of a single FOFP. Take two FOFPs as an example. Assume that the fiber core radius *r*_0_ and the fiber interval *a* in all the FOFPs are the same. As shown in Figure 8, the black dots represent the frequency of overlapping shift points of the first FOFP. The black line-covered area describes the first-order frequency aliasing of the first FOFP. The black crosses represent the frequency of overlapping points of the second FOFP, whose fiber arrangement direction is assumed to be 15° tilting from the first FOFP. The gray shade represents the overlapping spectrum from one of the six first-order overlapping frequency points. The value in the origin point of the frequency domain becomes extremely complex after aliasing.

**Figure 9:**
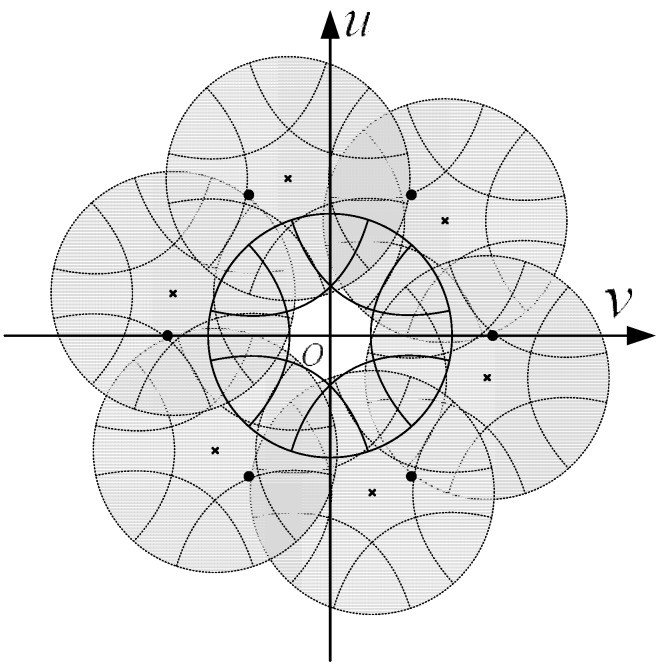
Frequency spectrum aliasing that is free from centroid error.

To avoid the aforementioned serious frequency spectrum aliasing, the highest frequency of the image spectrum from the previous FOFP must be less than the overlapping frequency of the next FOFP. An optical low-pass filter (OLPF) should be placed between two neighboring FOFPs. In practice, the reimaging process of an FOFP is more complicated than the description provided in Equation (16). Moreover, a certain amount of gap between two neighboring FOFPs is designed on purpose to defocus the image. This consideration somehow functions as an OLPF, and the frequency spectrum of the entering image can be limited within the overlapping frequency. The situation in which no systematic centroid error will be caused by multiple FOFPs in the intensifier is shown in Figure 9.

### 5.3. Systematic Error Reduction between the Output FOFP of the Intensifier and the Imaging Chip

The output FOFP is typically referred to as the optic taper coupling with the imaging chip. It transfers the enhanced image of the internal fluorescent screen to the CCD or CMOS chip behind it. Under the effect of the reimaging process (as shown in Equation (16)), the frequency spectrum of the image passing through the output FOFP lies within a circular area whose diameter *d*_freq_ is:

(43) $$d_{\text{freq}}=0.61/r_{0N}$$

where *r*_0*N*_ is the fiber core diameter of the output FOFP. The image passing through the output FOFP will be sampled by the imaging chip and transformed into digital signals. The pixels of a CMOS or CCD imaging sensor are typically arranged in rectangular grids. Assuming that the sampling interval of the imaging chip is *d*, inferring that the overlapping frequency is 1/*d* is easy. Similar to the analysis presented in Section 5.1, if no systematic error is caused by the imaging chip, then the spectrum radius that is being outputted from the intensifier should not exceed the overlapping frequency, which is as follows:

(44) $$\frac{1}{d}\geq d_{\text{freq}}$$

(45) $$d\leq \frac{r_{0N}}{0.61}\text{=}1.64r_{0N}$$

When the pixel size of the coupling imaging chip satisfies the constraint in Equation (45), the sampling frequency is sufficient to ensure that no systematic centroid error will occur in the imaging chips. 

## 6. Simulations and Experiments

Simulations and an experiment were conducted to verify the major conclusions drawn from the analysis of the reduction of FOFP-induced systematic centroid errors. Firstly, the simulation of a star spot entering at different places of an FOFP was presented, and the systematic centroid error distribution of the FOFP with respect to the entering position of the spot was compared with the expressions obtained in Section 5.1.2. Secondly, two simulations were performed to verify the constraint between the optical lens and the input FOFP of the intensifier. Thirdly, the calibration results of two intensified star tracker prototypes with different imaging chips were compared to demonstrate the analysis of systematic errors between the output FOFP of the intensifier and the imaging chip.

### 6.1. Simulation of the Systematic Centroid Error of an FOFP

A simulation of the systematic centroid error of an FOFP under different star spot positions was performed. The simulated parameter configuration of the star tracker is listed in Table 1. The simulation was conducted in a square FOFP area with 20 μm × 20 μm, and focused on a single fiber. The test star spots that were entering the FOFP were supposed to be Gaussian distributed, and their density was 0.2 μm in both directions. Following the FOFP model described in Section 4, integration and sampling were conducted on the original star spot and redistributed following the reimaging function.

**Table 1:** Simulation parameter configuration.

Parameters	Values
Fiber core diameter	5.5 μm
Fiber interval	6 μm
Star spot diameter	12 μm
Simulation area	20 μm × 20 μm
Test star spot density	0.2 μm

**Figure 10:**
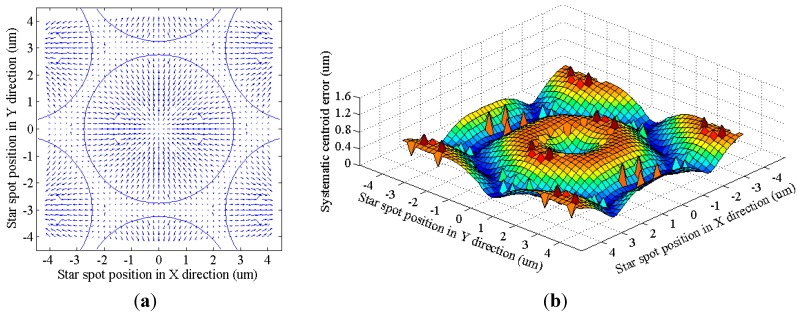
Systematic centroid error of the FOFP. (**a**) Error vector distribution; (**b**) Error magnitude distribution.

The simulation result is shown in Figure 10. Figure 10a presents the error vector distribution of the systematic error. The circles in the figure show where the fiber cores are. If the center of a star spot lies within the fiber cores, then the direction of the centroid error vector is radial. If the center of a spot falls within the border area of neighboring fibers, then systematic error decreases because of the counteracting effect. Figure 10b shows the error magnitude distribution. The maximum error can reach over 1 μm, which is obviously non-ignorable in high-accuracy star trackers. Moreover, the distribution pattern is approximately the same as the one deduced in the frequency domain (shown in Equations (29) and (30) and in Figure 7). The small bumps on the curved surface are caused by the boundary effect of the simulation area.

### 6.2. Simulation of the Match between the Optical Lens and the Input FOFP of the Intensifier

According to Equation (42), certain proportional relation between the circle of confusion of the lenses and the fiber interval of the FOFP must be satisfied. The following simulation kept all the values of the FOFP parameters unchanged while altering the circle of confusion. The systematic centroid error located at (0.8 μm, 1.4 μm) was observed through the changes. The chosen location is neither near the center of the fiber nor near the border area of fibers where the systematic centroid error is believed to be small, so it can represent the error magnitude better.

**Figure 11:**
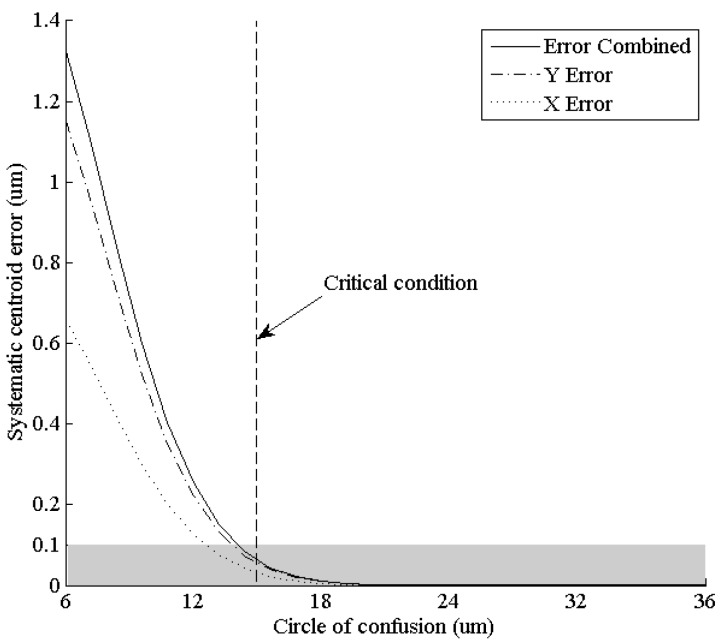
Systematic centroid error at (0.8 μm, 1.4 μm) under different circles of confusion.

As shown in Figure 11, the systematic error decreases rapidly as the circle of confusion of the entering star spot grows. If the circle of confusion is larger than the critical condition deduced from Equation (42) (14.94 μm), then systematic error magnitude will not exceed 0.1 μm (shaded area in the figure). The equivalent angular error is typically significantly less than 1 arc-second, and thus, it can be ignored. The simulation result verified that the circle of confusion of the optical lens should be 2.49 times that of the input FOFP fiber interval or more.

Not only the fiber interval but also the core-sheath ratio would affect the systematic centroid error. A simulation under different fiber core radii *r*_0_ was conducted, with all the other parameters unchanged in Section 6.1. The systematic centroid error located at (0.8 μm, 1.4 μm) was observed through the changes.

**Figure 12:**
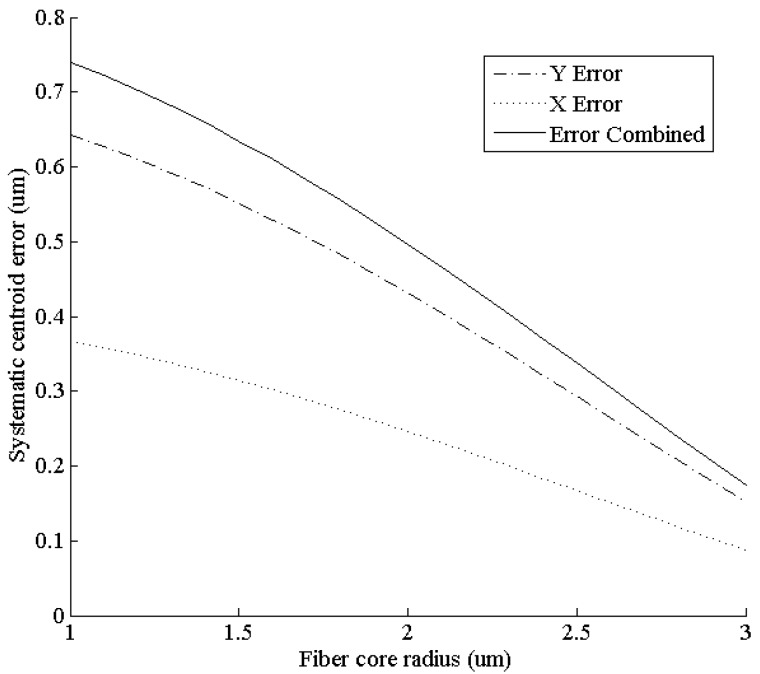
Systematic centroid error at (0.8 μm, 1.4 μm) under different fiber core radii.

As shown in Figure 12, the systematic error decreases as the fiber core radius grows. The decrease is nearly linear. When the fiber core radius reaches 3 μm (the upper limit caused by the fiber interval), the error magnitude is approximately half of the fiber core with a radius of 1 μm fiber core. That is, increasing the core-sheath ratio as much as possible under a fixed fiber interval will help harness systematic centroid error.

### 6.3. Experimental Investigation on the Match between the Output FOFP of the Intensifier and the Imaging Chip

Two prototypes of intensified star trackers were compared in terms of systematic centroid error. The imaging chips of the two star trackers are different. The first adopted a CCD chip from DALSA Inc. (Waterloo, ON, Canada), whereas the second chose a CMOS chip from CMOSIS Co (Antwerp, Belgium). The details are provided in Table 2. The pixel size of the CCD is half that of the CMOS, whereas both image sizes are 1 optical inch. Both the image chips were coupled with a same 2nd-generation double proximity image intensifier that was 18 mm in diameter. The output FOFP of the intensifier has a fiber core diameter of 5.5 μm and a fiber interval of 6 μm.

**Table 2:** Comparison of the two imaging chips.

Chip Name	FTT1010M	CMV4000
**Manufacturer**	Dalsa Inc.	CMOSIS Co.
**Chip Type**	Frame Transfer CCD	CMOS
**Pixel Size**	12 μm × 12 μm	5.5 μm × 5.5 μm
**Image Size**	1024 × 1024 pixels (12.288 mm × 12.288 mm)	2048 × 2048 pixels (11.264 mm × 11.264 mm)

**Figure 13:**
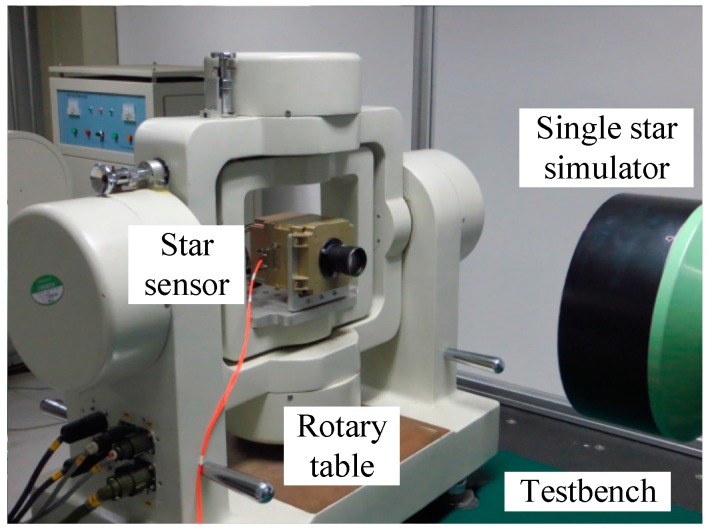
Laboratory calibration of the intensified star tracker.

The calibration residuals of both prototypes were processed to compare the systematic centroid error between the output FOFP of the intensifier and the imaging chip. As shown in Figure 13, the star tracker in calibration was installed on a two-axis rotary table that could generate arbitrary rotation angles and simulate a single star entering from different directions. To rule out the other two FOFP-induced systematic errors, both sensors adopted the same type of intensifiers and were calibrated with a same commercial optical lens. To eliminate the temporal random error caused by the gain variation of the MCP, centroid data were sampled 20 times in each point. Given the difference in spatial frequency, the low frequency parts of the residuals from the three-order polynomial fitting were abandoned to exclude the spatial random error caused by fiber arrangement defection.

**Figure 14:**
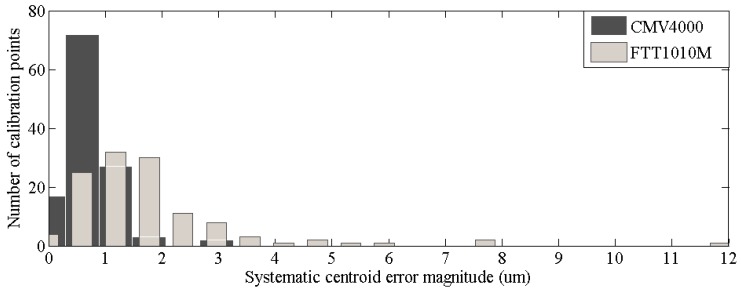
Histogram of systematic centroid errors.

The histogram of systematic centroid error magnitudes of the calibration points are shown in Figure 14. The error magnitudes of the calibration points from CMV4000 are significantly smaller than that from FTT1010M. Nearly all the error magnitudes of the points from CMV4000 is within 2 μm, whereas some of the calibration points from FTT1010M exceed 5 μm. According to Equation (45), given that the output FOFP of the fiber core diameter of the intensifier is 5.5 μm, pixel size should not exceed 4.5 μm to remain free from system centroid error. The pixel size of CMV4000 is obviously significantly closer to the set value than the pixel size of FTT1010M. The experiment result indicated that the systematic centroid error between the output FOFP of the intensifier and the imaging chip would be suppressed if the pixel size of the imaging chip matches with the fiber core diameter of the FOFP.

## 7. Conclusions

Based on the description of the imaging process of the optoelectronic detecting system of intensified star trackers, the error types caused by an FOFP were analyzed and classified. Three possible FOFP-induced systematic centroid errors can occur through the optical path, namely: (1) the error between the optical lens and the input FOFP; (2) the error among multiple FOFPs and (3) the error between the output FOFP and the imaging chip.

The expression of centroid errors in the frequency domain was presented. According to the expression, the centroid error only depends on the optical component that the star spot passes through. If the component is isoplanatic and invariant, then the centroid error is a constant bias; otherwise, the centroid error is different from place to place. Ground on the three-step image transmission process (integration, sampling and reimaging), the PSF and MTF of the FOFP were obtained.

To reduce FOFP-induced systematic centroid error, three potential error sources were analyzed. According to the analysis, two constraints and one suggestion were obtained: (1) The circle of confusion (6*σ*) should be 2.49 times or higher that of the fiber interval of the input FOFP to free the star tracker from the systematic errors caused by the input FOFP; (2) The pixel size of the imaging chip should not exceed 1.64 times that of the fiber core radius of the output FOFP; (3) Adding OLPF between neighboring FOFPs was recommended to reduce the systematic error caused by multiple FOFPs. Moreover, the exact expression of the systematic centroid error caused by the FOFP was deduced.

Finally, simulations and an experiment were conducted to verify the conclusions. The error distribution of a star spot that is entering the FOFP from different positions was simulated, and the result coincides with the systematic centroid expression of the FOFP. The relation between systematic error magnitude and the ratio of the circle of confusion of the optical lens to the fiber interval of the input FOFP was simulated, and showed that the systematic error would be less than 0.1 μm when the first constraint was satisfied. Moreover, the relation between the systematic error magnitude and the core-sheath ratio was simulated. The result demonstrated that the bigger the value of the core-sheath ratio, the less systematic it would be. The processed calibration residuals regarded as the systematic error between the output FOFP and the imaging chip of two intensified star tracker prototypes were compared. The prototype that adopted a 5.5 μm-pixel-size CMOS chip had considerably less systematic errors than the prototype that adopted a 12 μm-pixel-size CCD chip when the fiber core diameter of the output FOFP was 5.5 μm. The experiment result demonstrated the correctness of the second constraint.
